# Understanding the key processes of excellence as a prerequisite to establishing academic centres of excellence in Africa

**DOI:** 10.1186/s12909-020-02471-0

**Published:** 2021-01-07

**Authors:** Abebaw Fekadu, Claire Oppenheim, Tsegahun Manyazewal, Corey Nislow, Yimtubezinash Woldeamanuel, Asrat Hailu, Anteneh Belete, Dawit Wondimagegn, Charlotte Hanlon, Tsige Gebremariam, Asha Collins, Christopher J. Larson, Wondwossen Gebreyes, Eleni Aklilu, Adamson S. Muula, Sabina Mugus, Connor Caffery, Mirutse Giday, Getnet Yimer, Gail Davey, Girmay Medhin, Eyasu Makonnen

**Affiliations:** 1 Center for Innovative Drug Development and Therapeutic Trials for Africa (CDT-Africa), College of Health Sciences, Addis Ababa University, Addis Ababa, Ethiopia; 2 Global Health & Infection Department, Brighton and Sussex Medical School, Brighton, UK; 3 Department of Psychiatry, Boston University, School of Medicine, Boston, USA; 4 University of British Columbia, Vancouver, Canada; 5 College of Health Sciences, Addis Ababa University, Addis Ababa, Ethiopia; 6 King’s College London, Centre for Global Mental Health, Health Services & Population Research Department, Institute of Psychiatry, Psychology and Neuroscience, London, UK; 7 Department of Pharmaceutics and Social Pharmacy, College of Health Sciences, Addis Ababa University, Addis Ababa, Ethiopia; 8 Development, Aging & Regeneration Program, Sanford Burnham Prebys Medical Discovery Institute, San Diego, California USA; 9 The Global One Health initiative, Ohio State University, Columbus, OH USA; 10 Division of Clinical Pharmacology, Department of Laboratory of Medicine, Karolinska Institute, Stockholm, Sweden; 11 Department of Public Health, School of Public Health & Family Health, University of Malawi, Blantyre, Malawi; 12 Department of Pharmacology, Muhimbili University of Health and Allied Sciences, Dar Es Salaam, Tanzania; 13 Skaggs School of Pharmacy & Pharmaceutical Sciences, University of California San Diego, San Diego, California USA; 14 Aklilu Lemma Institute of Pathobiology, Addis Ababa University, Addis Ababa, Ethiopia; 15 Ohio State Global One Health Initiative, Office of International Affairs, the Ohio State University, Addis Ababa, Ethiopia

**Keywords:** Centre of excellence, Higher education, Africa, Excellence, Participatory leadership

## Abstract

**Background:**

Africa’s economic transformation relies on a radical transformation of its higher education institutions. The establishment of regional higher education Centres of Excellence (CoE) across Africa through a World Bank support aims to stimulate the needed transformation in education and research. However, *excellence* is a vague, and often indiscriminately used concept in academic circles. More importantly, the manner in which aspiring institutions can achieve academic excellence is described inadequately. The main objective of this paper is to describe the core processes of excellence as a prerequisite to establishing academic CoE in Africa.

**Methods:**

The paper relies on our collaborative discussions and real-world insight into the pursuit of academic excellence, a narrative review using Pubmed search for a contextual understanding of CoEs in Africa supplemented by a Google search for definitions of CoEs in academic contexts.

**Results:**

We identified three key, synergistic processes of excellence central to institutionalizing academic CoEs: participatory leadership, knowledge management, and inter-disciplinary collaboration. (1) Participatory leadership encourages innovations to originate from the different parts of the organization, and facilitates ownership as well as a culture of excellence. (2) Centers of Excellence are *future-oriented* in that they are constantly seeking to achieve best practices, informed by the most up-to-date and cutting-edge research and information available. As such, the process by which centres facilitate the flow of knowledge within and outside the organization, or knowledge management, is critical to their success. (3) Such centres also rely on expertise from different disciplines and ‘engaged’ scholarship. This multidisciplinarity leads to improved research productivity and enhances the production of problem-solving innovations.

**Conclusion:**

Participatory leadership, knowledge management, and inter-disciplinary collaborations are prerequisites to establishing academic CoEs in Africa. Future studies need to extend our findings to understand the processes key to productivity, competitiveness, institutionalization, and sustainability of academic CoEs in Africa.

## Background

The realization of the ambitious ‘Agenda 2063’ of the African Union, which sets out a 50-year vision (2013–2063) to place Africa at the centre of the global economy, relies on several factors, including the strategic transformation of higher education institutions across the continent [1–3]. The African Centres of Excellence (ACEs) project, supported by the World Bank, was initiated as a strategic input for transforming the African higher education landscape through addressing the critical shortage of skilled manpower in areas such as science, agriculture and health [4]. The Centre for Innovative Drug Development and Therapeutic Trials for Africa (CDT-Africa) was selected in 2016 as one of the ACEs in the health sector. We recognized that the Centre of Excellence (CoE) status was conferred on CDT-Africa to affirm its ambition to be a world-class centre of education and research that will improve access to medicines in Africa and thereby bring about sustainable health and economic growth. However, we were acutely aware that a significant amount of preparation and parallel development of supporting processes would be required to transform CDT-Africa into a regional CoE. Despite the challenge, this confirmation by the World Bank provided a substantial impetus and motivation for the desire to pursue excellence. In the process of pursuing the goal of excellence, the centre team was confronted with a series of conceptual and practical questions, including: What is a centre of excellence? How do we become a centre of excellence? What is the threshold for achieving the status of excellence? Who defines that status? How is that status maintained? Addressing these questions became even more challenging when, rather than defining a CoE in the abstract, we tried to provide a definition of a real-world regional center of excellence. The main aim of this paper is to share with readers what we identified as essential but often overlooked processes in the pursuit of academic excellence. We do not discuss in any detail the experience of CDT-Africa, although the information put forward and the conclusions drawn are inspired by reflection on that experience. The paper has three main sections. In the first section, a brief interpretation of excellence and a centre of excellence are provided. In the second section, we outline the processes critical for achieving excellence. In the last section, we discuss our experience and perspectives for creating a culture of excellence.

## Methods

The paper relies on our collaborative discussions and real-world insight into the pursuit of academic excellence, a narrative review, using PubMed search, for a contextual understanding of CoEs within Africa, supplemented with a Google search for the purposes of learning about the definitions of CoEs in academic contexts. As the primary approach, we have undertaken internal collaborative discussions in which the steps to be taken to transform CDT-Africa into a CoE were reflected upon. A research coordinator held numerous calls with the research team to understand the steps that were taken in implementing the CoE and to explore the main questions described above under the background. For the narrative review, the focus was on contextual understanding of CoEs and thus the team targeted papers published from Africa on the subject and indexed in PubMed between 2010 and 2020. The following search terms were used: Centre of excellence, Academic and Africa linked with the Boolean term AND. Additional Google search was used to obtain information on the definition of CoEs, the scope of operation, and to learn about the neglected processes of excellence.

## Results

Through the narrative review of publications from Africa, 162 titles were identified on the subject with 13 potentially relevant articles selected for full-text review. Of these, just two articles had discussed a CoE in any detail. Both articles focus on virtual CoEs. One of these articles describes a CoE on building capacity for biostatistics [5] and the second on a OneHealth approach for studying infectious diseases [6]. Two additional articles were identified through manual search.

### Defining a Centre of excellence

The term ‘excellence’ is widely used within academic and ‘specialist’ institutions, but this label has invited criticism, both from those who receive the status and from those who try to understand the implication. Part of the criticism likely arises from the fact that a clear definition is lacking. Michèle Lamont quotes an insightful, albeit strong, criticism. “The idea of excellence is ubiquitously evoked in academic contexts, yet little consensus exists concerning its meaning. As a term, it has the singular advantage of being entirely meaningless or to put it more precisely, non-referential” [7]. While quite harsh, this criticism should not encourage cynicism, but instead, spur efforts to better understand the term and apply it with increased rigor. Exploring the etymology of the word excellence may be a helpful initial step. The term excellence originates from the old French *excellent* meaning “outstanding, excellent,” and from the Latin *excellentem* meaning “towering, prominent, distinguished, superior, surpassing.” The root word excel comes from the Latin *excellere* in turn originating from *ex* (out of) _*+*_ *cellere* meaning to “surpass, be superior; to rise, be eminent, tower.” *Cellere* is also related to *celsus*-“high, lofty, great,” from Proto-Indo-European root *kel- (“to rise, be elevated, be prominent; hill”). The implication from these roots is clear: excellence refers to a relative status of prominence, superiority and uniqueness. The etymology also describes a word of *movement* and *growth*; excellence is not static [8, 9].

CoE relates to a team of specialised experts [10] or organisational environment or an entity [11] that is established to carry out outstanding research, education and training, provide leadership service and model best practice [10–12]. CoEs also can be considered as tools building specialised expertise [12] for innovation and service development pipeline [10].

CoEs exist in many fields, including business, technology, health services, research and academia, and are typically defined within the area of specialty based upon their specific organizational vision or mission. In this sense, a CoE is considered to have the specialty expertise that other institutions in the same field or domain lack. When we look beyond these mission-driven self-definitions and organizational components, and probe the values and processes that drive these CoEs towards their vision, commonalities emerge. It is thus apparent that it is not the components or structure of CoEs that make such centers truly “excellent,” but rather the underlying values and processes that both sustain them and drive them forward.

The University of California San Francisco (UCSF) Global Health Sciences Centers of Excellence Project offers perhaps one of the best definitions, one that does not require any comparison to other institutions in the same field. The CoE Project simply says that “Centers of excellence are change agents.” They further quote one leader of such a center in Uganda, who explains that, “Excellence is a moving target … [A center of excellence] is clearly that institution that is part of a global process constantly scanning the best way to do things, and then translating that excellence for the particular setting” [13].

Thus, a common mandate of CoEs implied by these definitions is the constant pursuit of progress, advancement and change. This pursuit of change and advancement may often lead to ‘disruption’, particularly at their inception. In the field of innovation, disruption describes two phenomena: (1) the origin of disruptive innovation is from “low end or new market footholds” and (2) disruptive trajectory, or the potential to overtake an established business or way of doing things. However, in the context of a centre of excellence, this mandate for change and disruption must be rooted with a commitment to do work of national and regional relevance. CoEs are so designated because of the potential national and regional impact of these centres.

Within academic spheres, CoEs may be understood in the context of the three types of university reforms identified by the European Commission [14]. (i) Horizontal reforms expand the institution through new academic programs. (ii) Integrative reforms are designed to enhance collaborations within and beyond the institutions. (iii) Vertical reforms are aimed at increasing ‘vertical differentiation’, which are designed to bring about improvements in quality and prestige. Although all three reforms have implication for a CoE, vertical reform is the best example of creating CoEs. Endowment of a CoE status confers prestige and empowers institutions to pursue quality, innovation and the conversion of knowledge into new technologies and solutions. The successful CoE maintains a unique profile while working to attain impact and world-class status. Academic CoE’s should harbour strong ambition to be top performers and to create an academic environment that can attract and retain talented students and researchers, that “… provides the foundation for dynamic society” [14].

### Foundations of excellence

**Fig. 1:**
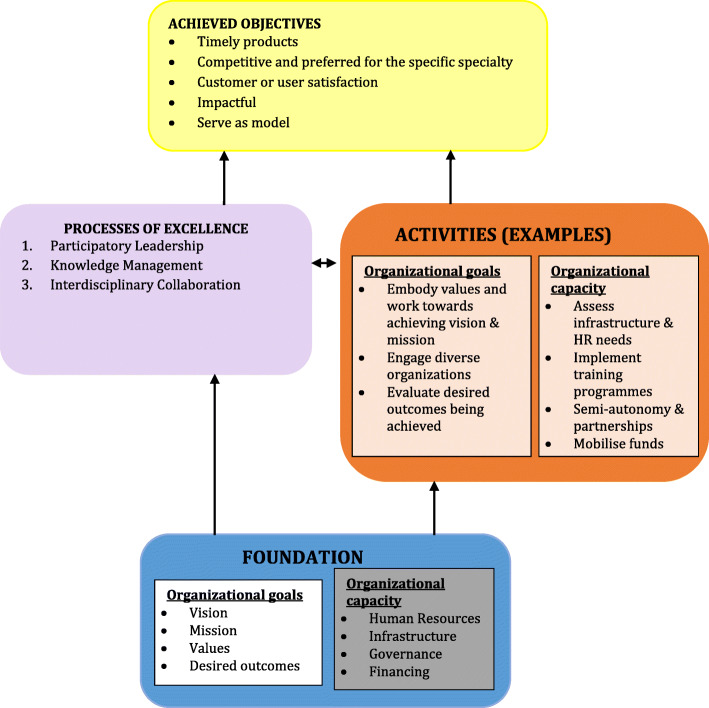
Foundation and progress towards achieving objectives of excellence

The foundations of excellence include organizational goals, organizational capacity and governance structure (Fig. 1). Organizational goals are reflected in the values, vision, mission and desired outcomes or objectives of the organization. The ambition of excellence - the desire to be a world-class institution in the area of interest - should be reflected in these goals. The goals of a CoE should be ‘extraordinary’ and may sometimes be referred to as ‘*moon shots’.* In addition to other needed infrastructure, it would be impossible for an organization to grow into a centre of excellence without administrative and technical/academic staff that embodies excellence. To sustain organizational capacity over the long term and provide the capacity to adapt to changes in the future operating environment, the centre should work diligently to ensure that financial sustainability is maintained. Finally, the governance structure of the CoE should allow sufficient autonomy and independence. This will enable the centre to be adequately self-directed and deliver quality products in a timely fashion.

One of the less apparent, but no less critical, features of CoEs is time management--the planning and the sense of urgency required to achieve organisational goals. Timely performance of scheduled tasks and timely delivery of results is of particular relevance to the process of achieving excellence [15]. If one single item was to represent excellence, it would be time--excellence cannot be achieved without delivering quality outcomes or products in a timely fashion. CDT-Africa’s assigned tasks are more complex and pressing and therefore demand efficient time management. We made conscious efforts to shape institutional cultures on effective use of time and we noticed resistance to such changes.

### Processes of excellence

**Fig. 2:**
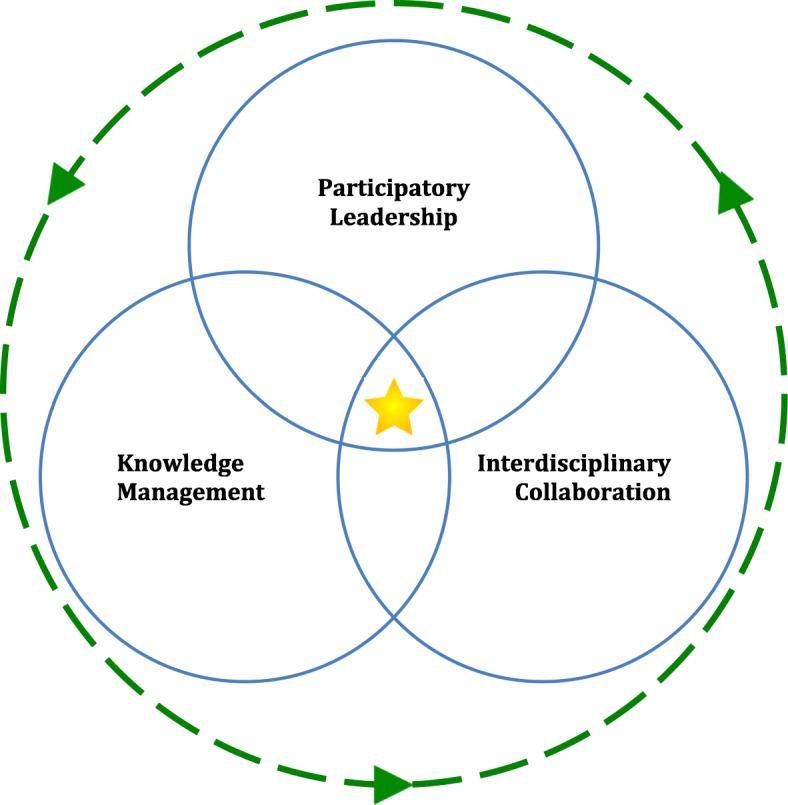
Three interactive processes of excellence

We distill the main processes of excellence into three key themes that we believe will drive a CoE to become an agent of change at a national, regional, and eventually global level: a true Center of Excellence. These three interactive processes of excellence (Fig. 2) are: Participatory Leadership, Knowledge Management and Interdisciplinary Collaboration. These processes will elevate a centre from a center of competency (a centre that is capable of doing things) to a Center of Excellence (a centre capable of delivering the best outcomes or results following the most efficient procedures).

### Participatory leadership

Participatory leadership, which is also known as democratic leadership, is a method and style of leadership in which team members are involved in guiding organizational goal-setting, decision-making, and problem-solving, as well as the processes to achieve these outcomes. This leadership model must solicit dissenting opinions and ensure that these opinions are respected and listened to without fear of retribution or ridicule. As well, CoE’s should ensure that all participatory leadership activities are rooted in the organization’s values, such as respect, innovation and diversity or equity. While the leader still retains decision-making authority, these decisions should be supported and validated by team members, thereby developing a sense of trust in and commitment to an organization that values their input. This encourages distributed accountability and a culture of excellence and innovation that emerges organically from diverse parts of the organization and not only from the main leadership.

Participatory leadership is contrasted with more traditional top-down, vertical or autocratic styles of leadership, in which subordinates have little to no say in the organizational direction and decision-making [16–19]. While well-suited for large, established institutions, autocratic leadership is not conducive to change, disruption and less likely to produce innovation.

### Importance of participatory leadership

A 2004 review of studies on research climate found that certain aspects of leadership were the primary drivers of research excellence. These leadership features included: group participation in leadership decisions, clear and coordinated objectives, visionary leadership and well-managed hiring and selection practices [20]. These leadership features and practices then facilitated quality research output.

Involving diverse organizational members and stakeholders in the leadership and decision-making process results in two key benefits: improved evidence-based decision-making and organizational buy-in. First, the individuals who carry out the day-to-day activities that move the organization towards its goals are likely to have valuable and practical insights into the best facilitators of and critical barriers to forward progress [18]. By involving those individuals in the discussion, efficient, viable, realistic and well-informed decisions can be made. Second, while traditional top-down governance is a “prescription for passive, uninvolved faculty [21],” in participatory leadership, members become more committed to an organization in which they feel involved and where their voices are valued during decision-making.

Participatory leadership also encourages the growth of future leaders, thereby helping ensure sustainable and smooth leadership transition. Continuity in leadership has been identified as a common strength of CoEs [22] and organizations which adopt participatory leadership styles, “can serve as leadership engines, fostering the development of new leaders committed less to protecting the organization from change than to putting the organization at the forefront of innovation” [21].

There are, however, a handful of potential disadvantages to participatory leadership, which can be addressed through thoughtful policies and mechanisms. A key downfall can be the time that it takes to engage in the participatory process and reach a decision. Consensus can take time, and we have already identified time as a key factor in excellence. Leaders must be prepared to enact streamlined, well-organized engagement mechanisms that can gather and synthesize input quickly and efficiently. They must also be prepared to make a final decision that is well informed, but not necessarily based upon complete consensus. Indeed, one could argue that complete consensus in not compatible with excellence and certainly not with innovation. Leaders must further ensure that members are fully aware of their ability to participate and have a clear understanding of the expectations and goals of their input [13]. Finally, members of the organization may feel pressure to conform to the rest of the group feedback or may fear retribution for expressing divergent thoughts or ideas. Therefore, leaders must ensure that members feel comfortable to express even dissenting opinions and that they believe their views will be both heard and respected [19, 21].

### Knowledge management

Knowledge management is one of the most central features of Centers of Excellence cited among the literature. Knowledge management refers to how an organization facilitates and directs the flow of knowledge both within itself, and between itself and external partners, collaborators, the public, the scientific community and other stakeholders. It is further critical that knowledge flows bidirectionally, obtaining information and input as well as disseminating knowledge. On a conceptual level, knowledge management also refers to an organization’s prioritization of commitment to both eliciting and disseminating information, knowledge and input from internal and external stakeholders alike [23, 24].

### Importance of knowledge management

Centers of Excellence are *future-oriented* in that they are constantly seeking to achieve best practices, informed by the most up-to-date and cutting-edge research and information available. As such, it is critical to emphasize the management of this knowledge.

Many health and research CoEs, especially clinical and translational research CoEs, are focused upon translating science into practice. Others go a step further to emphasize the critical linkages between education and training, research and practice. Within the context of CDT-Africa, education is especially important, and it must be built upon a foundation of human resource capacity in conducting world-class clinical and other researches. Strong knowledge management practices significantly improve the flow between theory, education, research and practice, thereby strengthening the link and closing the gap between research, innovation and practice [25].

As described above, knowledge management involves a bidirectional flow of knowledge (input and dissemination) both within and between an organization and its external partners and stakeholders. Externally, knowledge management ensures that organizations are able to stay at the forefront of best practices and new scientific advancements. It also ensures that organizations are actively contributing to this scientific culture, thereby elevating the organization’s status and visibility. Internally, knowledge management ensures that the organization is able to obtain critical feedback from its members, thereby facilitating effective and evidence-based improvement and strengthening members’ commitment to an organization in which their ideas and opinions are valued. It also ensures that all members of the organization are aware of opportunities, developments, successes and challenges.

Knowledge management entails “collaboration in the pursuit of knowledge or its application [21].” Sharing or managing internal and external knowledge throughout an organization necessarily means that many diverse, interdisciplinary members must be involved. When members effectively pool knowledge and use this knowledge to inform decision-making, organizational direction and scientific inquiry, the organization as a whole will be more effective and successful than any of its individual members [21].

For an organization to pursue excellence, it requires the ability to constantly evaluate itself and its surrounding scientific, academic, political, and social context and adjust accordingly. Therefore, knowledge management is crucial for successful participatory leadership, as leaders need to be effectively well informed in order to rapidly respond to and capitalize upon such information [22].

Knowledge management and participatory leadership also interact when it comes to the intellectual coherence of the organization. A ‘well-oiled’ organization cannot be just a collection of disparate projects linked by an organizational framework. The projects and research agendas should be scientifically coherent, and embody the overarching goal of excellence. Leaders may drive the intellectual direction of their organization but should do so in a way that is coherent and consistent with their members’ intellectual strengths and informed by intellectual challenges, a goal which requires both participatory leadership and knowledge management. Some CoEs have identified good dialogue between the organization and its leaders regarding strengths, challenges, bottlenecks and successes as a key to success and positive organizational culture [22].

### Interdisciplinary collaboration

The definition of interdisciplinary collaboration is fairly straightforward. It involves individuals and stakeholders from diverse disciplines, backgrounds, cultures and perspectives, coming together to solve a common problem or pursue common goals. The conceptualization of interdisciplinary should expand beyond strictly academic disciplines, to highlight wider inclusion of individuals such as non-academic community members, industry stakeholders, policymakers, funders and collaborators, as well as concerted efforts to ensure inclusion of women and minorities. While group consensus is not necessary for interdisciplinary collaboration to work, there must be an underlying foundation and culture of respect, sharing and willingness to work together and hear contradictory views.

### Importance of interdisciplinary collaboration

A 2001 study evaluated the Canadian Networks Centers of Excellence to understand what elements contributed to their culture of excellence. They found that, of the elements valued, the quality of scientific output was weighted at just 20 %, while programmatic values such as multidisciplinary and institutional diversity, research management, clear goal setting, and successful leverage of external networks were more important. Indeed, it is these underlying elements that drive excellence in scientific output downstream [26].

Regarding the importance of team-work, a 2006 study by the University of Illinois psychologists showed that teams of three to five individuals outperformed the best single individuals in letter-to-number coding problems. Teams, even those of a small number, are better than isolated individuals at viewing problems from diverse angles, generating multiple hypotheses and critically evaluating solutions.

Both intra-organizational and extra-organizational interdisciplinary collaboration are important. Within the organization, collaboration and communication has been shown to improve research productivity, and shared enthusiasm was more important than consensus [21]. Interdisciplinary collaboration within an organization also fosters intellectual synergy, as this allows for cross-fertilization of ideas, which can lead to further innovation and new, emerging areas of expertise [22]. Externally, diverse collaboration between an organization and its partners helps ensure that the organizational brand - and its excellence - continue to gain visibility, is engaged in the leading edge of the scientific community, and is open to the recognition and pursuit of more varied funding resources thereby supporting financial sustainability. However, these external partnerships need to be ‘active’ or ‘live,’ meaning that actual collaboration is ongoing, consistent and long-term. Partnerships and collaborations require continual attention, especially to protect the level of excellence of a particular CoE. Therefore, it may be prudent for an organization to ensure that they have enough partners and collaborators so as to expand their reach, but not too many that some partnerships become neglected.

Finally, collaboration between academics, researchers, community members and on-the-ground professionals is important for ensuring the applicability, access, and equity of scientific advancements. The involvement of those who the research intends to serve will help facilitate the translation of science into practice. Van de Ven [27] terms this as “engaged scholarship,” and defines it as “a participatory form of research for obtaining the different perspectives of key stakeholders (researchers, end-users, clients, sponsors, and practitioners) in studying complex problems. By involving others and leveraging their different kinds of knowledge, engaged scholarship can produce knowledge that is more penetrating and insightful than when scholars or practitioners work on the problem alone” [27].

We have previously discussed how interdisciplinary collaboration is a critical part of participatory leadership, in part because it drives the participatory aspect of this leadership and management style. Leaders must also set the tone and be the example of interdisciplinary collaboration within their organization. If the leaders preach this collaboration, but all leadership positions are held by individuals within the same or similar disciplines, with the same professional backgrounds, or are all the same gender, then their message of interdisciplinary collaboration loses validity.

With many different voices at the table, thoughtful management of this diverse knowledge and input becomes even more critical. The intended audience must inform how knowledge is elicited, disseminated, and generally managed. For example, how information is communicated to leaders of academic departments is likely to differ significantly from non-academic community leaders, or from industry stakeholders. However, the divisions between these different parties must still be permeable; there is a delicate balance between tailoring knowledge management to the audience, but still ensuring that everyone is at the same table. As Gunderman warns, “The higher and thicker the walls that separate an organization’s component divisions, the less effectively knowledge can be shared between them.” To effectively facilitate interdisciplinary collaboration and coordination, knowledge must be carefully and thoughtfully managed.

### Creating a “culture” of excellence

Nurturing a “culture of excellence” in an organization creates internal energy and harmony that drives the organization forward. This culture of excellence exists both in the outward, visible appearance and professional standards of the organization, as well as an underlying, communal culture of commitment to hard work and excellence.

### Appearance of professionalism

The physical appearance - both exterior and interior - of the actual buildings in which centers of excellence are housed can positively or negatively impact how both internal members and external persons perceive the CoE. As financial restrictions are an important and understandable consideration, spending money on appearances often may feel frivolous. However, it is undeniable that appearances do matter, especially when trying to convey an ethos of excellence. For example, when one enters a CoE, one should immediately feel a palpable sense that this is a place of quality and excellence. If so, external persons will feel that this is a place in which they would like to invest, build relationships, and one in which they can place their financial and intellectual trust. Internal organization members will feel proud to work there and committed to producing high-quality work that reflects the quality of their organization.

Here are a few key points that can contribute to this visible culture of excellence:

- Consistent use of organizational colors to provide continuity and make an impression of the organization’s “brand”
- Friendly, welcoming, well-trained and proficient administrative staff
- Welcoming, comfortable, uplifting reception/waiting area
- Promotional materials that are informative and demonstrate a sense of diverse, established and productive center activities
- Use of technology for scheduling and general organizational coordination
- Accessible Wi-Fi is appreciated by all
- Consistent door labeling system demonstrates professionalism and facilitates accessibility of organization members
- A clearly marked restroom helps put visitors at ease and prevents them from having to ask potentially embarrassing questions
- Open office doors suggesting an active, collaborative and productive culture
- Sense of cultural and national pride is demonstrated through artwork. The organization feels warm and intimate, rather than clinical and impersonal.
- Demonstration of technological advancement and savvy

### Culture of commitment to excellence

We have also described how an organization must have an underlying, internal culture of commitment to hard work and excellence. We suggest that achieving this pervasive level of commitment requires **bidirectional** relationship and investment between the organization and the individual.

#### Organization ➔ individual

The organization must demonstrate clear, consistent and long-term investment in its individual members:

- Advocate, support and fight for the individual
- Appropriately incentivize the individual
- Involve individuals in decision-making and goal-setting
- Prioritize and invest in efficient infrastructure, including salary, purchasing, and other elements of financial system/management
- Clear and transparent communication of organization plans, directions, objectives to individuals
- Stable, accessible and transparent leadership

#### Individual ➔ organization

If the organization demonstrates the above investment in the individual, then the individual will, in turn, feel committed to work hard for the organization:

- Individual feels committed to the organization in which they can see a clear path towards career advancement and opportunity
- Individual wants to continue working for organization in the long-term (retention of talent)
- Individual wants to work hard for organization
- Individual feels valued by organization
- Individual feels respected by organization
- Individual wants to collaborate with others in the organization, rather than being secretive and protective of individual work and reputation
- Individual feels like they are part of an organizational community, as well as connected to a larger community in which they are having a measurable impact

Several limitations have to be considered. The paper was based primarily on evaluation of internal processes, which may lead to “myopic” and experiential view. This may limit generalizability of the conclusions. However, these views are based on the experience of a large number of experts who contributed to this paper. We have that the paper would offer important clues to those aspiring to establish a CoE, or are in the early stages of establishing a CoE. It may also be useful for academic leaders and even government officials who have ambitions to initiate CoEs. Additionally, the review has focused on publications from Africa. However, we have consulted CoEs of diverse industries and organisations.

## Conclusion

The fact that defining and understanding excellence is a challenge or that excellence is too lofty a goal should not deter any organization to aim for excellence. In fact, this paper is an attempt to persuade organizations, particularly higher education institutions, to aim to establish CoEs knowing that with a commitment to developing strong foundations, partnerships, processes and perseverance, any institution can become a centre of excellence. It is not the conceptual embrace of excellence that matters-the ‘fruit’ of excellence will show by the centre’s leading by example as evidenced by quality of the students, the innovative products developed and the consumer satisfaction achieved. We propose participatory leadership, knowledge management, and inter-disciplinary collaborations as prerequisites to establishing academic CoEs in Africa. Future studies need to extend our findings to understand the processes key to productivity, competitiveness, institutionalization, and sustainability of academic CoEs in Africa.

## Data Availability

Not applicable.

## Abbreviations

ACE: Africa Centre of Excellence
CDT-Africa: Centre for Innovative Drug Development and Therapeutic Trials for Africa
CoE: Centre of Excellence
UCSF: University of California, San Francisco
