# Correction to: Understanding the key processes of excellence as a prerequisite to establishing academic centres of excellence in Africa

**DOI:** 10.1186/s12909-021-02523-z

**Published:** 2021-02-10

**Authors:** Abebaw Fekadu, Claire Oppenheim, Tsegahun Manyazewal, Corey Nislow, Yimtubezinash Woldeamanuel, Asrat Hailu, Anteneh Belete, Dawit Wondimagegn, Charlotte Hanlon, Tsige Gebremariam, Asha Collins, Christopher J. Larson, Wondwossen Gebreyes, Eleni Aklilu, Adamson S. Muula, Sabina Mugus, Conor R. Caffrey, Mirutse Giday, Getnet Yimer, Gail Davey, Girmay Medhin, Eyasu Makonnen

**Affiliations:** 1 Center for Innovative Drug Development and Therapeutic Trials for Africa (CDT-Africa), College of Health Sciences, Addis Ababa University, Addis Ababa, Ethiopia; 2 Global Health & Infection Department, Brighton and Sussex Medical School, Brighton, UK; 3 Department of Psychiatry, Boston University, School of Medicine, Boston, USA; 4 University of British Columbia, Vancouver, Canada; 5 College of Health Sciences, Addis Ababa University, Addis Ababa, Ethiopia; 6 King’s College London, Centre for Global Mental Health, Health Services & Population Research Department, Institute of Psychiatry, Psychology and Neuroscience, London, UK; 7 Department of Pharmaceutics and Social Pharmacy, College of Health Sciences, Addis Ababa University, Addis Ababa, Ethiopia; 8 Development, Aging & Regeneration Program, Sanford Burnham Prebys Medical Discovery Institute, San Diego, California USA; 9 The Global One Health initiative, Ohio State University, Columbus, OH USA; 10 Division of Clinical Pharmacology, Department of Laboratory of Medicine, Karolinska Institute, Stockholm, Sweden; 11 Department of Public Health, School of Public Health & Family Health, University of Malawi, Blantyre, Malawi; 12 Department of Pharmacology, Muhimbili University of Health and Allied Sciences, Dar Es Salaam, Tanzania; 13 Center for Discovery and Innovation in Parasitic Diseases, Skaggs School of Pharmacy and Pharmaceutical Sciences, University of California San Diego, La Jolla, California USA; 14 Aklilu Lemma Institute of Pathobiology, Addis Ababa University, Addis Ababa, Ethiopia; 15 Ohio State Global One Health Initiative, Office of International Affairs, the Ohio State University, Addis Ababa, Ethiopia

**Correction to: BMC Med Educ 21, 36 (2021)**

**https://doi.org/10.1186/s12909-020-02471-0**

Following publication of the original article [1], the authors identified an error in the author name of Conor R. Caffrey and also in his affiliation.

The incorrect author name is: Connor Caffery

The correct author name is: Conor R. Caffrey

The incorrect author affiliation is: Skaggs School of Pharmacy & Pharmaceutical Sciences, University of California San Diego, San Diego, California, USA.

The correct author affiliation is: Center for Discovery and Innovation in Parasitic Diseases, Skaggs School of Pharmacy and Pharmaceutical Sciences, University of California San Diego, La Jolla, California, USA

The original article has been corrected.
